# Effect of Surveillance Method on Reported Characteristics of Lyme Disease, Connecticut, 1996–2007

**DOI:** 10.3201/eid1802.101219

**Published:** 2012-02

**Authors:** Starr-Hope Ertel, Randall S. Nelson, Matthew L. Cartter

**Affiliations:** Connecticut Department of Public Health, Hartford, Connecticut, USA

**Keywords:** epidemiologic methods, data collection, population surveillance, endemic diseases, public health practice, Lyme disease, Connecticut, bacteria, vector-borne infections

## Abstract

To determine the effect of changing public health surveillance methods on the reported epidemiology of Lyme disease, we analyzed Connecticut data for 1996–2007. Data were stratified by 4 surveillance methods and compared. A total of 87,174 reports were received that included 79,896 potential cases. Variations based on surveillance methods were seen. Cases reported through physician-based surveillance were significantly more likely to be classified as confirmed; such case-patients were significantly more likely to have symptoms of erythema migrans only and to have illness onset during summer months. Case-patients reported through laboratory-based surveillance were significantly more likely to have late manifestations only and to be older. Use of multiple surveillance methods provided a more complete clinical and demographic description of cases but lacked efficiency. When interpreting data, changes in surveillance method must be considered.

## Abstract 2

The epidemiology of Lyme disease varies by surveillance method.

Lyme disease, a multisystem disease caused by the spirochete bacterium *Borrelia burgdorferi*, is the most commonly reported vector-borne disease in Connecticut and in the United States (*1*). During 1996–2007, Connecticut contributed 33,457 (15%) cases to the national surveillance case count, with a mean of 83.4 cases per 100,000 population reported annually, and consistently led the nation in reported annual incidence rate during the study period (*2*). Diagnosis of Lyme disease is based on clinical findings, serologic evidence of infection, and history of exposure to *Ixodes scapularis* ticks. Early stages of illness are most readily diagnosed by identification of erythema migrans. Later-stage illness can involve the musculoskeletal, neurologic, or cardiovascular systems. Positive serologic results are necessary for identifying and classifying patients with later manifestations.

During 1996–2007, the Connecticut Department of Public Health (CDPH) received Lyme disease reports through 4 surveillance methods: passive physician, active physician, enhanced laboratory, and mandatory laboratory. Physician-based surveillance (passive and active) was conducted during the entire study period and relied on health care providers to report new diagnoses of Lyme disease. Active surveillance comprised a voluntary network of health care providers who reported cases 1× per month. Enhanced laboratory surveillance, conducted during 1996–1997, required participating Connecticut laboratories to send supplemental case report forms with each positive *B. burgdorferi* result to the ordering physician. In January 1998, to study the effectiveness of a newly released Lyme disease vaccine, mandatory laboratory surveillance was implemented that required all laboratories to report positive and equivocal results to CDPH. Follow-up, conducted by CDPH staff, involved sending a letter and supplemental report form to the ordering physician. To assist the physician, demographic and patient-identifying information from the laboratory report was incorporated into the form. Mandatory laboratory surveillance ended after 2002 when the Lyme disease vaccine was removed from the market. In 2007, mandatory reporting of positive Lyme disease results was reinstated for laboratories with electronic reporting capability. Two large commercial laboratories provided electronic reports. Follow-up was reestablished by using the previous method, i.e., CDPH staff sent a letter and supplemental report form to the ordering physician.

Public health surveillance methods for infectious diseases change over time, depending on program priorities and resources, advancements in diagnostic testing, modifications to surveillance case definitions, and changing reporting modalities (e.g., electronic laboratory reporting). Lyme disease surveillance data provide a measure of the relative geographic distribution of this disease and its effect on public health in Connecticut and have been used to assess the effectiveness of control and prevention activities (*3**–**5*). These data also form part of the risk communication messages provided to the general public, advocacy groups, media, political leaders, health care providers, and public health professionals. We examined how surveillance method affected the classification of reported clinical and demographic characteristics of case-patients and the incidence of Lyme disease in Connecticut, during 1996–2007.

## Materials and Methods

### Surveillance Case Definition

Lyme disease reports were categorized by using the national surveillance case definition issued in 1996 (*6*). A case was defined as 1) physician report of erythema migrans of >5 cm in diameter or 2) at least 1 objective late manifestation (i.e., musculoskeletal, neurologic, or cardiovascular) with laboratory confirmation of infection with *B. burgdorferi* by enzyme immunoassay, immunofluorescent assay, or Western immunoblot. CDPH classified reports that did not meet the case definition as not a case. Because clinical information is required for case classification, when supplemental follow-up reports were not returned, they were considered lost to follow-up. The distinct report forms used for each surveillance method contained the following data elements: case-patient demographic characteristics (sex, age, race, ethnicity), clinical findings (erythema migrans or late manifestations), seasonality, and case status. Seasons were defined as winter (December–February), spring (March–May), summer (June–August), and fall (September–November).

### Data Collection

The statewide Lyme disease surveillance system was maintained by an average of 1.5 full-time employees. Reports were entered into the National Electronic Telecommunications System for Surveillance (NETSS), a public health surveillance information system that used Epi Info 6.0 (*7*). In 2007, NETSS and Microsoft Excel (Microsoft, Redmond, WA, USA) were used. Supplementary variables necessary for follow-up and maintenance of reports were added to the standard NETSS variables by CDPH staff and included health care provider name and contact information, license number, and origin of report.

A potential case could have been reported through >1 surveillance method. For consistency in classification, the origin of case reports was entered in the following hierarchy: active surveillance, passive surveillance, enhanced laboratory surveillance, and mandatory laboratory surveillance. Data were cleaned and duplications were removed at the end of each year.

### Data Analyzed

The following statistics were calculated across surveillance methods: annual mean number of reports and cases, annual incidence rates, proportion of reports by case status, demographic characteristics of case-patients, seasonality of cases, and clinical and laboratory findings. Incidence per 100,000 population was determined by using decennial census data covering the year of data collection (1990 or 2000). Statistical tests were performed by using Epi Info 6.0. We used χ^2^ test with the Yates continuity correction. A p value <0.05 was considered significant. The positive predictive value (PPV) of reports of potential cases was calculated for each type of surveillance method by determining the ratio of cases to reports.

## Results

### Overall Analysis

During 1996–2007, CDPH staff processed 87,174 Lyme disease reports, of which 7,278 (8.3%) were duplicate entries and were removed from the database. A total of 79,896 individual reports were analyzed. Of these, 43,767 (54.8%) were reported through mandatory laboratory surveillance, 19,350 (24.2%) through passive physician surveillance, 13,040 (16.3%) through active physician surveillance, and 3,739 (4.7%) through enhanced laboratory surveillance. Overall, 33,457 (41.9%) reports were classified as cases, and 26,318 (32.9%) as not cases; 20,121 (25.2%) were lost to follow-up (Table 1). Except for calculation of PPV, reports classified as lost to follow-up were excluded from further analyses.

**Table 1:** Number of Lyme disease reports, by status and surveillance method, Connecticut, 1996–2007*

Status	PS	AS	ELS	MLS	Total
Case	12,185	8,666	1,949	10,657	33,457
Not a case	4,962	4,316	1,783	15,257	26,318
Lost to follow-up	2,203	58	7	17,853	20,121
Total†	19,350	13,040	3,739	43,767	79,896

*PS, passive physician surveillance 1996–2007; AS, active physician surveillance 1996–2007; ELS, enhanced laboratory surveillance 1996–1997; MLS, mandatory laboratory surveillance 1998–2002 and 2007. †Positive predictive values: PS, 63.0; AS, 66.5; ELS, 52.1; MLS, 24.3; total, 41.9.

**Figure 1:**
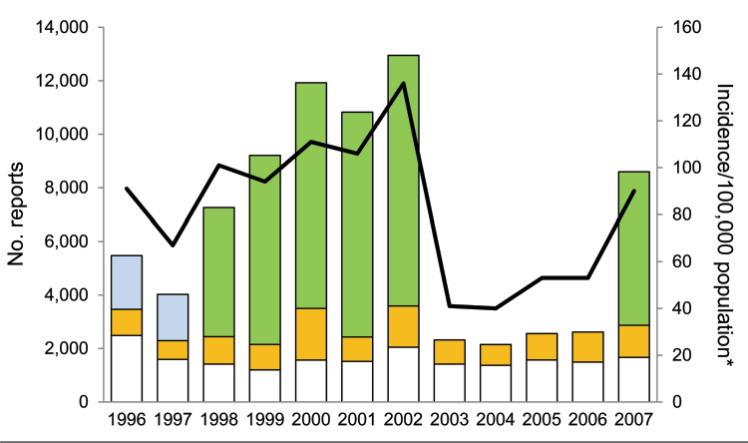
Number of Lyme disease surveillance reports received and incidence per 100,000 population, Connecticut, 1996–2007. White bar sections, passive surveillance; gold bar sections, active surveillance; blue bar sections, enhanced laboratory surveillance; green bar sections, mandatory laboratory surveillance; line, incidence, determined by using decennial census data encompassing the year data were reported.

During 1996, Connecticut had 5,473 reports of Lyme disease. The number of reports increased with the successive implementation of enhanced and mandatory laboratory surveillance reporting, peaking at 12,947 in 2002 (Figure 1). In 1998, the first year of mandatory laboratory surveillance, the overall number of Lyme disease reports increased by 80.3%, cases increased by 49.4%, and physician-based reporting increased by 26.9% over the previous year; incidence was 104.5 cases per 100,000 population. In 2003, the first year after laboratory reporting ended, the overall number of reports decreased by 82.1%, cases decreased by 69.7%, and physician-based reporting decreased by 37.8% over the previous year; incidence was 41.2 cases per 100,000 population. During 2003–2006, the period with no laboratory surveillance, the number of total reports dropped substantially to an annual mean of 2,411, a 78.5% decrease from the 1999–2002 annual mean. In 2007, laboratory surveillance was reinstated for laboratories with the capability to electronically report results. The total number of reports increased by 228.3%, the number of cases increased by 71.0% over the previous year (Figure 1), and the incidence nearly doubled to 89.8 cases per 100,000 population. An average of 16.0% more cases were reported through physician-based surveillance during years with mandatory laboratory reporting.

The PPV varied across surveillance methods and was highest for physician-based surveillance methods (Table 1). Less than 25% of reports received through mandatory laboratory surveillance were classified as cases. Cases reported through this method accounted for nearly one third (31.9%) of all cases during the study period.

### Demographic Characteristics

**Figure 2:**
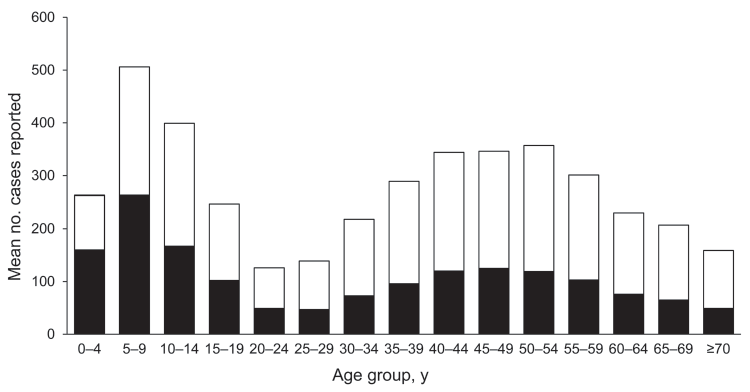
Mean annual number of Lyme disease cases, by age group and surveillance method, Connecticut, 1996–2007. Black bar sections, physician-based surveillance; white bar sections, laboratory-based surveillance.

The median age of case-patients was 38 years (range 34–43 years). Case-patients <20 years of age were more likely to be reported through physician-based surveillance (p<0.001); laboratory-based surveillance was more likely to report case-patients >40 years of age (p<0.001) (Figure 2). Overall, whites accounted for 82.0% of cases, similar to the state’s racial distribution, and the distribution did not differ significantly by surveillance method. Ethnicity data were available for approximately one third (32.6%) of case-patients; only 1.2% were reported as Hispanic. Laboratory-based surveillance reported an average of 32 Hispanic case-patients annually, compared with 20 reported through physician-based surveillance (Table 2). On average, 9.6% more male than female case-patients were reported by each surveillance method.

**Table 2:** Demographic characteristics of Lyme disease case-patients, by surveillance method, Connecticut, 1996–2007*

Characteristic†	No. (%) case-patients				
	PS, n = 12,185	AS, n = 8,666	ELS, n = 1,949	MLS, n = 10,657	Total, n = 33,457
Sex					
M	6,707 (55.0)	4,690 (54.1)	1,128 (57.9)	5,761 (54.1)	18,286 (54.7)
F	5,453 (44.8)	3,969 (45.8)	814 (41.8)	4,856 (45.6)	15,092 (45.1)
Unknown	25 (0.2)	7 (0.1)	7 (0.4)	40 (0.4)	79 (0.2)
Race					
White	10,402 (85.4)	6,772 (78.1)	1,440 (73.9)	8,811 (82.7)	27,425 (82.0)
Black	83 (0.7)	47 (0.5)	14 (0.7)	119 (1.1)	263 (0.8)
Asian/Pacific Islander	83 (0.7)	34 (0.4)	10 (0.5)	63 (0.6)	190 (0.6)
American Indian/Alaska Native	18 (0.1)	7 (0.1)	1 (0.1)	6 (0.1)	32 (0.1)
Other	83 (0.7)	17 (0.2)	1 (0.1)	32 (0.3)	133 (0.4)
Unknown	1,516 (12.4)	1,789 (20.6)	483 (24.8)	1,626 (15.3)	5,414 (16.2)
Ethnicity					
Hispanic	202 (1.7)	33 (0.4)	3 (0.2)	156 (1.5)	394 (1.2)
Non-Hispanic	7,051 (57.9)	884 (10.2)	16 (0.8)	2,550 (23.9)	10,501 (31.4)
Unknown	4,932 (40.5)	7,749 (89.4)	1,930 (99.0)	7,951 (74.6)	22,562 (67.4)

*PS, passive surveillance; AS, active surveillance; ELS, enhanced laboratory surveillance; MLS, mandatory laboratory surveillance. †Median ages: PS, 34 y; AS, 36 y; ELS, 43 y; MLS, 42 y; total, 38 y.

### Clinical Features

Of the 33,457 cases, 66.2% were characterized by erythema migrans only, 27.6% by >1 late manifestation and positive laboratory findings, and 6.2% by both (Table 3, Table 4). Overall, symptoms of erythema migrans only were more likely to be reported through physician-based surveillance than through laboratory-based surveillance (75.8% vs. 50.3%; p<0.001). Conversely, late manifestations were more likely to be reported through laboratory-based surveillance than through physician-based surveillance (43.2% vs. 18.1%; p<0.001). Of all case-patients reported through laboratory-based surveillance, 30.5% had Lyme arthritis, compared with 13.4% of those reported through physician-based surveillance. Of cases characterized by late manifestations only, arthritis was most frequently reported (72.1%). Of late manifestation cases for which arthritis was reported, 74.0% were based on physician surveillance and 70.8% on laboratory surveillance (p<0.001). Laboratory-based surveillance was more likely to report case-patients with second- or third-degree atrioventricular block (p = 0.051).

**Table 3:** Clinical manifestations of Lyme disease, by surveillance method, Connecticut, 1996–2007*

Clinical manifestations	No. (%) cases				
	PS, n = 12,185	AS, n = 8,666	ELS, n = 1,949	MLS, n = 10,657	Total, n = 33,457
Erythema migrans only	9,489 (77.9)	6,324 (73.0)	1,032 (53.0)	5,305 (49.8)	22,150 (66.2)
Late manifestations only	2,059 (16.9)	1,725 (19.9)	763 (39.1)	4,678 (43.9)	9,225 (27.6)
Both	637 (5.2)	617 (7.1)	154 (7.9)	674 (6.3)	2,082 (6.2)

*PS, passive surveillance; AS, active surveillance; ELS, enhanced laboratory surveillance; MLS, mandatory laboratory surveillance.

**Table 4:** Late manifestations of Lyme disease, by surveillance method, Connecticut, 1996–2007*

Manifestation†	No. cases (%)				
	PS, n = 2,059	AS, n = 1,725	ELS, n = 763	MLS, n = 4,678	Total, n = 9,225
Lyme arthritis	1,448 (70.3)	1,353 (78.4)	532 (69.7)	3,318 (70.9)	6,651 (72.1)
Bell palsy	454 (22.1)	259 (15.0)	143 (18.7)	814 (17.4)	1,670 (18.1)
Radiculoneuropathy	117 (5.7)	131 (7.6)	84 (11.0)	501 (10.7)	833 (9.0)
Lymphocytic meningitis	61 (3.0)	35 (2.0)	19 (2.5)	108 (2.3)	223 (2.4)
Encephalitis/encephalomylitis	38 (1.5)	18 (1.0)	26 (3.4)	116 (2.5)	198 (2.1)
Second- or third-degree heart block	40 (1.9)	26 (1.5)	10 (1.3)	58 (1.2)	134 (1.4)

*PS, passive surveillance; AS, active surveillance; ELS, enhanced laboratory surveillance; MLS, mandatory laboratory surveillance. †May have included >1 late manifestation per case. Percentages do not add to 100%.

### Seasonality

In 72.9% of cases, illness onset occurred during the summer (76.3% physician-based vs. 66.8% laboratory-based cases) (Table 5). Erythema migrans occurred in 84.2% of cases with onset during the summer. Erythema migrans was significantly more likely to be reported during the summer through physician-based surveillance than through laboratory-based surveillance (71.3% vs. 28.7%; p<0.001). Late manifestations were 2× more likely to be reported through laboratory-based surveillance during the summer months (17.5% vs. 8.2%; p<0.001).

**Table 5:** Lyme disease cases, by clinical manifestation, season, and surveillance method, Connecticut, 1996–2007

Clinical manifestation/season	No. (%) cases				
	PS, n = 10,535*	AS, n = 8,281*	ELS, n = 1,767*	MLS, n = 8,685*	Total, n = 29,268
All					
Winter	278 (2.6)	244 (2.9)	76 (4.3)	548 (6.3)	1,146 (3.9)
Spring	1,029 (9.8)	923 (11.1)	154 (8.7)	1,118 (12.9)	3,224 (11.0)
Summer	8,121 (77.1)	6,234 (75.3)	1,276 (72.2)	5,707 (65.7)	21,338 (72.9)
Fall	1,107 (10.5)	880 (10.6)	261 (14.8)	1,312 (15.1)	3,560 (12.2)
Erythema migrans					
Winter	84 (0.8)	56 (0.7)	7 (0.4)	73 (0.8)	220 (0.8)
Spring	741 (7.0)	624 (7.5)	34 (1.9)	425 (4.9)	1,824 (6.2)
Summer	7,315 (69.4)	5,500 (66.4)	993 (56.2)	4,164 (47.9)	17,972 (61.4)
Fall	739 (7.0)	515 (6.2)	78 (4.4)	501 (5.8)	1,833 (6.3)
Total	8,879 (84.3)	6,695 (80.8)	1,112 (62.9)	5,163 (59.4)	21,849 (74.7)
Late manifestations					
Winter	194 (11.7)	188 (11.9)	69 (10.5)	475 (13.5)	926 (3.2)
Spring	288 (17.4)	299 (18.8)	120 (18.3)	693 (19.7)	1,400 (4.8)
Summer	806 (48.7)	734 (46.3)	283 (43.2)	1,543 (43.8)	3,366 (11.5)
Fall	368 (22.2)	365 (23.0)	183 (27.9)	811 (23.0)	1,727 (5.9)
Total	1,656 (15.7)	1,586 (19.2)	655 (37.1)	3,522 (40.6)	7,419 (25.3)

*PS, passive surveillance; AS, active surveillance; ELS, enhanced laboratory surveillance; MLS, mandatory laboratory surveillance.

## Discussion

In Connecticut, data obtained through 4 surveillance methods during 1996–2007 demonstrated that the epidemiology of Lyme disease is subject to variation by surveillance method. The number of reports, proportion of reports classified as cases, incidence, and demographic and clinical characteristics of case-patients differed between physician-based and laboratory-based surveillance. Although some of the annual fluctuation in reports and cases might be attributable to an actual increase or decrease in disease, the substantial changes seen indicate that the principal factor most likely resulted from changes in surveillance method over time. As these surveillance artifacts show, changes in surveillance methods can cause changes in trends. Therefore, the nature of the surveillance method and the effect of changes in the method are necessary to consider when interpreting the data.

Lyme disease surveillance methods ultimately rely on physicians to report the necessary clinical information to classify cases. Because health care providers in outpatient settings often underreport commonly seen illnesses (*8**,**9*), in Connecticut, follow-up for mandatory laboratory surveillance might help serve as a reminder system for physicians to report cases. This fact could explain the 16.0% increase in the average annual number of cases reported through physician-based surveillance during years when laboratory surveillance was mandatory.

Our data showed that physician-based surveillance, combined with laboratory-based surveillance, resulted in more comprehensive clinical and demographic information and higher incidence of illness than each method alone. Of all reported cases, nearly one third (31.9%) originated through laboratory-based surveillance. However, use of laboratory-based surveillance is inefficient: only 24.3% were classified as cases. Case-patients reported through laboratory-based surveillance also differed significantly in age groups, reporting older case-patients; clinical information, reporting more late stage illness; and seasonal data, reporting more cases during the fall and winter months. Our combined surveillance methods contributed to, and broadened, the overall epidemiologic description of Lyme disease in Connecticut.

This study has several limitations. First, laboratory-based surveillance was difficult to evaluate independently of physician-based surveillance. Because health care professionals could potentially report by using each of the surveillance methods, we used a hierarchy to help reduce bias toward 1 surveillance method over another. Second, because active surveillance providers volunteered to participate, these physicians were more likely to be those most interested in Lyme disease surveillance. Therefore, physicians who volunteered to participate in active surveillance might have been more likely to report cases in a strictly passive surveillance system.

To satisfy the sometimes conflicting goals of surveillance methods and allocation of public health resources, collection of case data needs to be streamlined. Two potential alternatives may be the following: modeling by using sampling schemes or greater use of electronic information systems, which is planned in Connecticut. Electronic laboratory reporting, automation of follow-up requests, and Web-based provider reporting will conserve resources, and provide incident data information demanded of public health agencies.

When determining which methods of Lyme disease surveillance to use, the purpose of that surveillance and available resources need to be considered. In Lyme disease–endemic states where the epidemiologic purpose might primarily be to monitor geographic, clinical, and demographic trends, intensive statewide surveillance is not essential. Rather, surveillance needs to be conducted consistently over time. Intensive surveillance efforts may even be counterproductive when not sustainable because of limited resources or when resources are diverted from other public health activities. Replacing traditional case-reporting surveillance methods with less labor-intensive data collection methods, such as regular population-based surveys, may be suitable for following trends and estimating disease (*10*).
